# Efficacy and Safety of Radiotherapy and Systemic Treatments in Adrenocortical Carcinoma: Systematic Review and Meta-Analysis

**DOI:** 10.1210/clinem/dgaf457

**Published:** 2025-08-11

**Authors:** Anita Pfeffer, Nóra Beke, Dorottya Bakó, Márk Hernádfői, Tamás Kói, András Fogarasi, Andrea Párniczky, Péter Hegyi, Miklós Garami

**Affiliations:** Pediatric Center, Tűzoltó Street Department, Semmelweis University, Budapest 1094, Hungary; Centre for Translational Medicine, Semmelweis University, Budapest 1085, Hungary; Centre for Translational Medicine, Semmelweis University, Budapest 1085, Hungary; Centre for Translational Medicine, Semmelweis University, Budapest 1085, Hungary; Centre for Translational Medicine, Semmelweis University, Budapest 1085, Hungary; Department of Neurology, Bethesda Children’s Hospital, Budapest 1146, Hungary; Centre for Translational Medicine, Semmelweis University, Budapest 1085, Hungary; Department of Stochastics, Institute of Mathematics, Budapest University of Technology and Economics, Budapest 1111, Hungary; Centre for Translational Medicine, Semmelweis University, Budapest 1085, Hungary; Department of Neurology, Bethesda Children’s Hospital, Budapest 1146, Hungary; Centre for Translational Medicine, Semmelweis University, Budapest 1085, Hungary; Department of Pulmonology, Heim Pál National Pediatric Institute, Budapest 1089, Hungary; Institute of Pancreatic Diseases, Semmelweis University, Budapest 1085, Hungary; Centre for Translational Medicine, Semmelweis University, Budapest 1085, Hungary; Institute of Pancreatic Diseases, Semmelweis University, Budapest 1085, Hungary; Institute for Translational Medicine, Medical School, University of Pécs, Pécs 7624, Hungary; Pediatric Center, Tűzoltó Street Department, Semmelweis University, Budapest 1094, Hungary; Centre for Translational Medicine, Semmelweis University, Budapest 1085, Hungary

**Keywords:** adrenocortical carcinoma, cytotoxic chemotherapy, irradiation, mitotane, radiotherapy, targeted therapy

## Abstract

**Context:**

Adrenocortical carcinoma (ACC) is a rare, aggressive malignancy with a high recurrence and mortality rate even after complete resection. Therefore, intensification of adjuvant therapies is crucial, although their effectiveness remains controversial.

**Objective:**

This study aimed to determine the efficacy and safety of available treatments by considering the prognostic factors affecting disease outcomes.

**Methods:**

The search was conducted across 3 databases (PubMed, Embase, and CENTRAL) in October 2024. Eligible studies compared overall survival (OS) and recurrence-free survival (RFS) in patients with ACC treated with and without systemic (mitotane, cytotoxic chemotherapy) or localized (radiotherapy) nonsurgical treatments. In total, 86 studies met the inclusion criteria for the systematic review, and 62 for the meta-analysis. Two reviewers extracted data independently. Adjusted hazard ratios (aHR) with 95% CI were calculated for survival outcomes.

**Results:**

Mitotane significantly improves OS (aHR: 0.54; CI: 0.41-0.70) and RFS (aHR: 0.60; CI: 0.43-0.84), even in stage I-III disease (OS aHR: 0.71; CI: 0.52-0.98; RFS aHR: 0.65; CI: 0.50-0.85). Radiotherapy showed a trend toward improved OS (aHR: 0.66; CI: 0.38-1.15) and RFS (aHR: 0.65; CI: 0.35-1.23), with significant benefits in stage I-III (OS aHR: 0.68; CI: 0.50-0.93; RFS aHR: 0.71; CI: 0.63-0.81). The impact of cytotoxic chemotherapy on OS remains uncertain (aHR: 0.61; CI: 0.08-4.78).

**Conclusion:**

Mitotane significantly improves survival outcomes in patients with ACC, while radiotherapy exhibits potential benefits, particularly in localized disease. Further research is needed to verify the efficacy of cytotoxic chemotherapy, and randomized controlled trials are required to provide robust evidence of different treatment approaches.

Adrenocortical carcinoma (ACC) is a rare and aggressive malignancy, with an annual incidence estimated at 0.7 to 2 cases per million individuals (1, 2). Despite improvements over time in the clinical management of ACC, its prognosis remains largely unfavorable (1, 3) due to its aggressive biological behavior (4) and limited therapeutic options (5). The 5-year survival rate ranges from 15% to 40% (6); however, there is significant heterogeneity in individual outcomes (7) influenced by stage, surgical margin, and hormone secretion of the tumor (7, 8).

The only potentially curative treatment is surgical removal of the primary tumor with regional lymph node dissection (4). Nevertheless, more than 50% of patients experience recurrence, even after complete R0 resection (9, 10), leading to a poor prognosis. Treatment options for inoperable or metastatic disease are limited (11). Therefore, intensification of adjuvant therapies is an unmet clinical need to prevent disease recurrence and improve survival outcomes (1, 3, 12).

Available therapies include mitotane, radiotherapy, cytotoxic chemotherapy, and targeted therapies (13). Mitotane, an adrenolytic agent, has been widely used in clinical practice since 1960 (14, 15). It is administered alone or in combination with cytostatic chemotherapy in adjuvant and palliative care settings (16). However, the effectiveness of this treatment shows conflicting outcomes (13, 17) and significant side effects (18, 19). Evidence for adjuvant radiotherapy is even more limited than for mitotane, and the role of adjuvant chemotherapy remains poorly defined (20).

Due to the rarity of ACC, prospective randomized studies are limited (21), and there is a need for level I evidence to guide clinical practice and decision-making about adjuvant therapies (8).

This meta-analysis aimed to comprehensively assess the impact and tolerability of current therapeutic modalities on overall survival (OS) and recurrence-free survival (RFS) in patients with ACC, considering prognostic factors that affect disease outcomes.

## Materials and Methods

We utilized the Preferred Reporting Items for Systematic Reviews and Meta-Analyses (PRISMA) statement to present the results of our research (Table S1) (22). We adhered to the methodological guidance outlined in the Cochrane Handbook (23, 24). Our study protocol was registered on PROSPERO (CRD42023475740), and we fully adhered to the protocol.

### Search Strategy and Selection Criteria

On November 15, 2023, we performed a comprehensive search of 3 scientific databases: PubMed, Embase, and Cochrane Central Register of Controlled Trials (CENTRAL). We updated our search on October 18, 2024. In addition, we screened reference lists of eligible articles using “citationchaser” to identify all pertinent studies (25). Our search key focused on domains of adrenocortical carcinoma and various treatment approaches ((“*adrenocortical*” OR (“*adrenal*” AND cort*) AND (carcinoma* OR cancer*)) AND (“*therapy*” OR “treatment” OR “*chemotherapy*” OR “*adjuvant*” OR “*radiotherapy*” OR “*irradiation*” OR “*radiation*” OR “*mitotan*” OR “*mitotane*” OR (“*targeted*” AND “*therapy*”) OR (“*personalized*” AND “*therapy*”) OR “*immunotherapy*” OR “cisplatin*” OR “*doxorubicin*” OR “etoposid*”)). No language or further restrictions were applied during the systematic search.

After automatic and manual removal of duplicates with the assistance of reference management software (EndNote X9), 2 reviewers independently screened publications by title, abstract, and full text according to predefined eligibility criteria. Inter-rater agreement during selection was evaluated using Cohen's kappa coefficient. Any conflicts in selection were resolved by a third author.

Randomized controlled trials (RCT) and observational studies were included in the analysis. Conference abstracts reporting relevant data were also included in the analysis. Eligible articles compared the outcomes of patients with ACC receiving systemic (mitotane, cytotoxic chemotherapy, immunotherapy, targeted therapy) or localized (radiotherapy) nonsurgical therapy to those who did not. There were no restrictions on the age or extent of the disease in the patients enrolled. The primary outcomes of the eligible studies involved the assessment of survival measures, such as OS, RFS, and local recurrence-free survival (LRFS). These studies reported on survival duration or proportion in patients with ACC and presented results using unadjusted or adjusted Cox hazard models.

Articles examining the effectiveness of investigated therapies based on factors such as stage, surgical margin, hormone secretion, and serum mitotane levels were also included. In addition, we considered secondary outcomes related to treatment side effects.

Case reports, case series, review articles, and studies without comparison groups were excluded.

### Data Extraction

Two independent reviewers (A.P. and N.B.) extracted data from eligible studies using a predefined Excel datasheet (Office 365, Microsoft, Redmond, WA, USA). An independent third reviewer (D.B.) resolved any disagreements. Data extracted included first author, publication year, digital object identifier, study design, study duration, sample size in each comparison group, demographic data, outcome measures such as hazard ratios (HR) for adjuvant therapy with corresponding 95% CI in terms of RFS and OS derived from univariate or multivariate Cox regression analyses, along with survival durations and associated CIs, potential prognostic factors adjusted for in multivariate Cox regression model, and follow-up times.

### Risk of Bias and Quality of Evidence Assessment

Two independent review authors (A.P. and N.B.) evaluated the risk of bias according to the Cochrane Collaboration recommendation: the Risk of Bias-2 tool (26) was employed for RCTs, the ROBINS-I tool (27) for non-randomized, and QUIPS (28) for prognostic studies. Any disagreements were resolved by involving a third investigator (D.B.).

We used the Grading of Recommendations Assessment, Development, and Evaluation (GRADE) approach (29) and GRADEpro software to assess the quality of evidence in our findings.

### Statistical Analyses

To take into account the differences in the circumstances of the studies, we included random effect terms in all the analyses.

To pool the HRs, we used classical inverse variance random-effects meta-analysis on the logarithms of the HRs with the REML tau estimator and Hartung–Knapp adjustment. We visualized the pooled outcomes and their 95% CIs on forest plots. Due to the low number of studies involved in the analysis, we did not provide prediction intervals. Heterogeneity was assessed by calculating the (univariate) *I^2^* measure and its CI and performing the Cochrane Q test. Subgroup analyses based on stage, hormone secretion, and surgical margin were also performed.

In observational studies, the unadjusted HR can be misleading due to potential confounders; for example, certain treatments may be administered to patients with more severe conditions. Therefore, we pooled unadjusted and adjusted HRs separately. We pooled adjusted HRs (aHRs) from the multivariate Cox regression together with HRs of studies using matching-based adjustment. As randomization avoids confounding, HRs of RCTs were included in the adjusted analyses. The studies involved used similar variables for the adjustments. Nevertheless, as differences in the covariates used may cause heterogeneity, following the approach of Renehan et al (30) for odds ratios, we created a table showing the covariates involved in the adjustment for each study.

Except for studies utilizing well-established large databases (National Cancer Database [NCDB] and Surveillance, Epidemiology, and End Results [SEER]), when clearly identifiable and significant overlap in patient populations was observed, the study with the smaller sample size was excluded. However, in cases where overlap was not suspected but could not be definitively determined, the studies were retained.

The handling of publications utilizing the NCDB or SEER database required careful consideration. These databases cover an extensive timeframe; different studies used different subperiods and patient inclusion criteria, leading to the varying extents of overlapping timeframes. Consequently, retaining only the largest study would result in significant loss of patient data. Using all the studies with a multivariate meta-analysis approach was also not feasible since the strength of the correlations between the published HRs presumably differs substantially depending on the extent of the overlaps. Instead of the multivariate approach, to minimize selection bias, we conducted several sensitivity analyses using different nonoverlapping study sets. The analysis with the highest number of patients for a specific outcome was considered the primary result and presented in the results section of the manuscript. The results from the sensitivity analysis with a smaller sample size for the corresponding primary result are provided in the Supplemental Material (22).

A few involved HRs were calculated by digitizing the Kaplan–Meier curves with the WebPlotDigitizer tool (31).

Publication bias analyses were conducted for the main outcomes when at least 10 studies were available (32). Publication bias was visually assessed using funnel plots and formally evaluated using Egger's test.

A leave-one-out sensitivity analysis was performed for the main outcomes to explore the contribution of individual studies to overall heterogeneity.

Statistical analyses were performed using packages “IPDfromKM” (v0.1.10), “meta” (v6.2.1), “metaSurvival” (v0.1.0), and “survival” (v3.3.1) of the R statistical software (version 4.2.1.). A *P* value of less than 0.05 was considered significant for all statistical analyses.

## Results

### Study Selection

**Figure 1.**
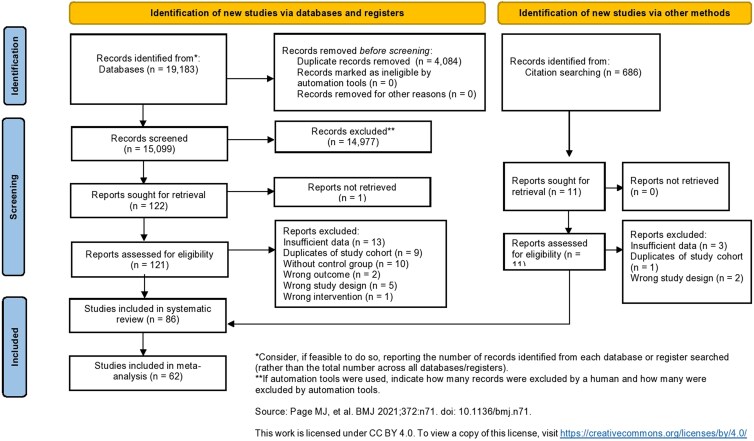
PRISMA flowchart presenting the selection process of studies included in the systematic review and meta-analysis.

The systematic search yielded 19 183 articles. Of these, 86 met the eligibility criteria for inclusion in the systematic review, and 62 were included in the meta-analysis. The PRISMA flowchart presents the selection process in Fig. 1 (33).

### Characteristics of the Included Studies

The studies analyzed consisted of cohort studies and 2 RCTs. The basic characteristics are summarized in Table S2 (22). The geographical distribution of the included studies is illustrated in Fig. S1 (22).

Due to the limited number of articles comparing side effects of different therapies, conducting a meta-analysis on adverse effects was not feasible.

A summary of the findings from the systematic review is presented in Table S3 (22).

### Effect of Mitotane on Overall and Recurrence-Free Survival

**Figure 2.**
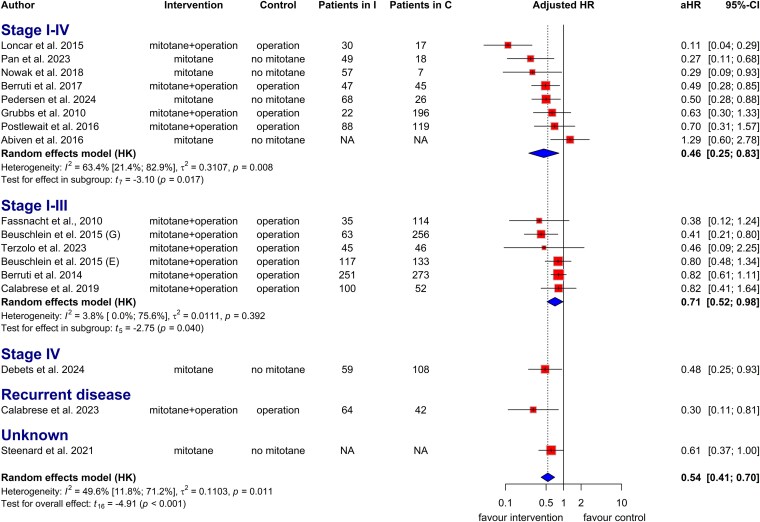
Forest plot with pooled adjusted hazard ratio (HR), representing the risk of death in patients treated with and without mitotane based on disease stage. Abbreviations: aHR, adjusted hazard ratio; NA, not applicable.

Fifteen articles provided univariate and 16 multivariate HRs for mortality risk in patients treated with mitotane. This therapy was significantly associated with prolonged OS in both unadjusted (HR: 0.62, CI: 0.45 to 0.85; heterogeneity *I*^2^: 63%; Fig. S2) (22) and adjusted analyses (aHR = 0.54, CI: 0.41 to 0.70; *I*^2^: 50%; Fig. 2).

In stage I to III patients, adjuvant mitotane significantly reduced the risk of death in multivariate analysis (aHR = 0.71, CI: 0.52 to 0.98; *I^2^*: 4%; Fig. 2). Univariate analysis revealed a similar trend without statistical significance (HR = 0.80, CI: 0.43 to 1.49; *I^2^*: 40%; Fig. S2) (22).

Significantly improved survival was observed in patients who achieved a target plasma mitotane concentration of 14 mg/L compared to those who did not, based on both univariate (HR = 0.46, CI: 0.27 to 0.80; *I*^2^: 58%; Fig. S3) (22) and multivariate results (aHR = 0.47, CI: 0.33 to 0.67; *I*^2^: 0%; Fig. S4) (22). No significant difference in the effectiveness of mitotane on OS between hormone-producing and non-hormone-producing subgroups was observed (HR = 0.62, CI: 0.14-2.63; *I^2^*: 61%; Fig. S5) (22).

Adjuvant mitotane demonstrated a tendency to improve survival in patients who underwent R0 resection (HR = 0.70, CI: 0.42 to 1.17; *I^2^*: 61%; Fig. S6) (22).

**Figure 3.**
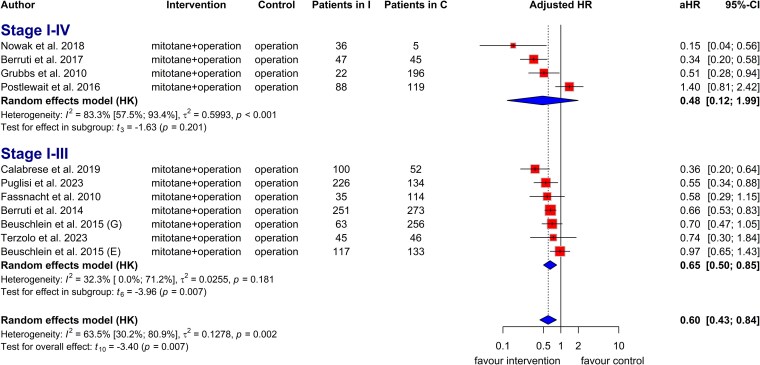
Forest plot with pooled adjusted hazard ratio, representing the risk of tumor recurrence in patients treated with and without mitotane treatment based on disease stage. Abbreviation: aHR, adjusted hazard ratio.

A total of 9 articles reported unadjusted and 10 reported adjusted HRs on the risk of tumor recurrence in patients treated with adjuvant mitotane. Its use was significantly associated with prolonged RFS in adjusted analysis (aHR = 0.60, CI: 0.43 to 0.84; *I^2^*: 64%; Fig. 3). A similar trend was observed in the unadjusted analysis at the border of statistical significance (HR = 0.72, CI: 0.48 to 1.06; *I^2^*: 75%; Fig. S7) (22).

Adjuvant mitotane significantly improved RFS in stages I to III based on multivariate analysis (aHR = 0.65, CI: 0.50 to 0.85; *I^2^*: 32%; Fig. 3). Univariate analysis showed a similar trend without statistical significance (HR = 0.79, CI: 0.51 to 1.22; *I^2^*: 55%; Fig. S7) (22).

It prolonged RFS in both the hormone-producing and non-hormone-producing subgroups (HR = 0.47, CI: 0.21 to 1.03; *I^2^*: 55%; Fig. S8) (22) and also significantly improved RFS in patients who underwent R0 resection (HR = 0.72, CI: 0.58-0.89; *I^2^*: 0%; Fig. S9) (22).

### Effect of Radiotherapy on Overall, Recurrence-Free, and Local Recurrence-Free Survival

**Figure 4.**
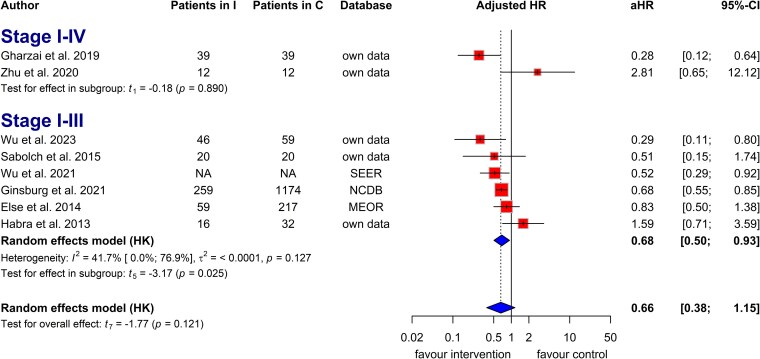
Forest plot with pooled adjusted hazard ratios illustrates the risk of death in patients treated with adjuvant radiotherapy vs those who underwent surgery without radiotherapy, stratified by disease stage. Abbreviations: aHR, adjusted hazard ratio; MEOR, Michigan Endocrine Oncology Repository; NA, not applicable; NCDB, National Cancer Database; SEER, Surveillance, Epidemiology, and End Results.

The analysis of 8 studies that reported adjusted HRs for OS in patients with ACC indicated that adjuvant radiotherapy provided a clinically relevant benefit compared to surgery alone. This result was not statistically significant due to 2 outlier studies with few participants (aHR = 0.66, CI: 0.38 to 1.15; Fig. 4). A similar result was observed in the analysis of 9 studies investigating OS in patients treated with and without radiotherapy (aHR = 0.76, CI: 0.47 to 1.22; Fig. S10) (22). Adjuvant radiotherapy improved OS in patients with ACC, with an odds ratio (OR) of 2.03 (CI: 0.60 to 6.84; *I^2^*: 49%; Fig. S11) (22).

In the adjusted analysis, adjuvant radiotherapy significantly increased OS in patients with stage I to stage III disease compared with surgery without radiotherapy (aHR = 0.68, CI: 0.50 to 0.93; *I^2^*: 42%; Fig. 4). A similar trend was seen without statistical significance in the unadjusted analysis (HR = 0.79, CI: 0.50 to 1.27; *I^2^*: 37%; Fig. S12) (22) and the evaluation of OS in patients treated with and without radiotherapy based on adjusted HRs (aHR = 0.77, CI: 0.50 to 1.20; *I^2^*: 46%; Fig. S13) (22).

The effect of radiotherapy on OS in patients with stage IV disease was assessed across 3 studies reporting univariate HRs. Although we observed a clinical benefit in prolonging OS among stage IV patients, statistical significance was not reached due to high heterogeneity (HR = 0.59, CI: 0.21 to 1.69; *I^2^*: 60%; Fig. S14) (22).

The results of the sensitivity analysis for OS in radiotherapy-treated patients are presented in the Supplementary Material (Figs. S15-S29) (22).

Our results support the efficacy of adjuvant radiotherapy in improving OS in patients who underwent R1 resection (Fig. S30) (22).

**Figure 5.**
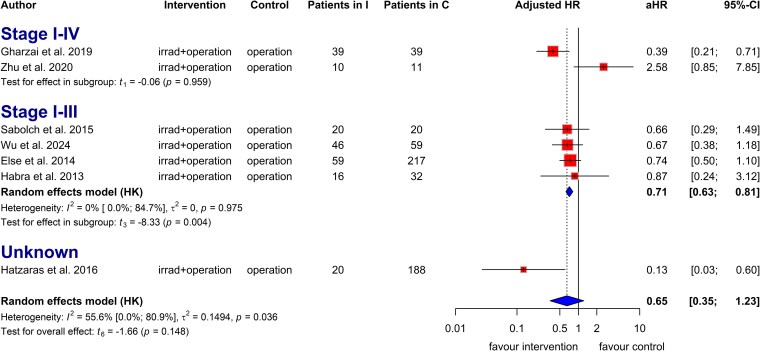
Forest plot with pooled adjusted hazard ratio, representing the risk of tumor recurrence in patients treated with and without radiotherapy based on disease stage. Abbreviation: aHR, adjusted hazard ratio.

Seven studies reported adjusted HRs for tumor recurrence risk in patients with ACC treated with adjuvant radiotherapy. Adjuvant radiotherapy provided a clinical benefit in prolonging RFS, although without statistical significance due to high heterogeneity (aHR = 0.65, CI: 0.35 to 1.23; *I^2^*: 56%; Fig. 5). Patients who received adjuvant radiotherapy showed higher odds of RFS than those who did not (OR: 1.89, CI: 0.75 to 4.75; *I^2^*: 17%; Fig. S31) (22). Adjuvant radiotherapy significantly improved RFS in patients with stage I to III disease (aHR = 0.71, CI: 0.63 to 0.81; *I^2^*: 0%; Fig. 5).

Five articles presented multivariate HRs for LRFS in patients with ACC treated with adjuvant radiotherapy, showing a clear clinical benefit in prolonging LRFS without statistical significance due to high heterogeneity and small sample size (aHR = 0.55, CI: 0.16 to 1.90; *I^2^*: 56%; Fig. S32) (22). The pooled analysis showed a preference for adjuvant radiotherapy in improving LRFS, with an OR of 4.10 (CI: 0.89 to 18.87; *I^2^*: 68%; Fig. S33) (22).

### Effect of Adjuvant Cytotoxic Chemotherapy, Chemoradiotherapy, and Systemic Therapy on Overall Survival

**Figure 6.**
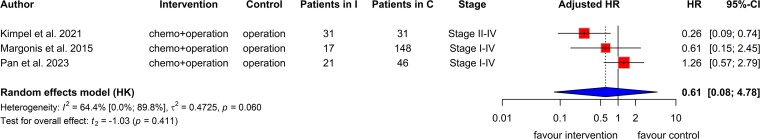
Forest plot with pooled adjusted hazard ratio, representing the risk of death in patients treated with and without cytotoxic chemotherapy. Abbreviation: aHR, adjusted hazard ratio.

Three studies examined the effect of adjuvant cytotoxic chemotherapy on OS. The adjusted analysis suggested a potential clinical benefit in reducing the risk of mortality, but this finding lacked statistical significance (aHR = 0.61, CI: 0.08 to 4.78; *I^2^*: 64%; Fig. 6). Furthermore, the wide CI caused by an outlier study published by Pan et al (7) should be considered when interpreting this finding.

Adjuvant chemoradiotherapy prolonged OS without statistical significance (aHR = 0.78, CI: 0.44 to 1.36; *I^2^*: 8%; Fig. S34) (22).

Systemic therapy significantly improved OS (aHR = 0.74, CI: 0.57 to 0.97; *I^2^*: 0%; Fig. S35) (22).

### Risk of Bias Assessment, Certainty of Evidence, and Assessment of Heterogeneity

Of the articles, 6% showed a low, 54% moderate, and 40% high risk of bias, primarily due to confounding factors such as missing adjustments for age and stage (Figs. S36-S38) (22).

As most of the included studies were observational, the levels of certainty were generally very low based on the findings and comprehensive assessment of the evidence (Table S4) (22).

During the assessment of publication bias, we found that in all but one case, the Egger test did not detect a significant small-study effect. For the adjusted mitotane OS HR, the *P* value was slightly below 0.05 (*P* = .0397). Considering that no significant small-study effect was found for the adjuvant mitotane RFS HR, whose meta-analysis included a slightly different set of studies, we found no strong evidence for a small-study effect overall (Fig. S39-S41) (22).

Most analyses showed statistical heterogeneity due to underlying differences in study design and patient populations, as well as small sample sizes and imbalances between intervention and control groups. In univariate analyses, heterogeneity was likely largely attributable to differences in patient characteristics and the absence of adjustment for prognostic factors. Subgroup analysis revealed that, among studies performing multivariate analysis to investigate the effect of radiotherapy, small-sample institutional studies contributed disproportionately to heterogeneity compared to large database studies (Figs. S42-S44) (22). Additionally, leave-one-out analyses revealed that certain individual studies investigating the effect of mitotane on overall survival had a notable influence on heterogeneity. This pattern was not observed in studies evaluating recurrence-free survival outcomes related to adjuvant mitotane or in the radiotherapy subgroup. Furthermore, the pooled effect size did not change substantially in any of the analyses (Figs. S45-S48) (22).

## Discussion

Translational science plays a key role in translating research findings into routine medical practice (34, 35). This systematic review and meta-analysis addresses a gap by evaluating the efficacy and safety of radiotherapy and available systemic therapies for ACC, considering the prognostic factors that influence disease outcomes.

Mitotane is the primary adjuvant therapy in ACC (36); however, its use remains controversial, and predictors of response are lacking (37). Two previous meta-analyses have examined its effect in an adjuvant setting: both found a significant benefit for OS (38, 39), while only one showed a significant improvement in RFS (38). Our meta-analysis demonstrated that mitotane significantly improves OS and RFS, reducing the risk of death and tumor recurrence by 47% and 40%, respectively.

Current guidelines recommend adjuvant mitotane after ACC resection for patients at a high risk of recurrence (stage III-IV, R1 resection, or Ki-67 > 10%). Nevertheless, for patients at low or moderate risk (stage I-II, R0 resection, or Ki-67 ≤ 10%), its use should be discussed individually (36, 39). The ADIUVO trial, the first RCT comparing adjuvant mitotane therapy to surveillance in resected ACC patients with low to intermediate risk (stage I-III, R0 resection, Ki67 ≤ 10%), reported no significant benefit of adjuvant mitotane in improving RFS or OS (40). However, this study was limited by the small sample size, and the recurrence rate was much lower than expected. Our subgroup analysis revealed that adjuvant mitotane significantly prolonged OS and RFS in patients with stage I to III disease. Nevertheless, it is important to emphasize that we were unable to adjust for other key prognostic factors, such as R status or Ki67, that influence disease outcomes. As a result, we could not clearly differentiate between low- and high-risk groups concerning recurrence risk. Some of the patients included in our analysis might have been at higher risk of tumor recurrence, which could explain the more pronounced benefit of adjuvant mitotane observed in our study compared to its effect in low-risk patients.

According to the GRADE assessment (29), the certainty of evidence for the effect of mitotane on OS and RFS—both in the overall population and the subgroup with stage I to stage III—was rated as very low, mainly due to the retrospective nature of the included studies and lack of adjustment for key clinical variables in several analyses. These factors substantially reduce confidence in the estimated effect sizes, highlighting the need for prospective, well-controlled studies to better clarify the role of mitotane in ACC treatment.

Recent guidelines emphasize reaching mitotane blood levels above 14 mg/L (39). Our findings support this recommendation and show that achieving this serum level significantly prolongs OS. Based on the GRADE assessment (29), the certainty of evidence for this association was rated as moderate.

Hormone secretion by the tumor, especially hypercortisolism, is associated with poor prognosis (41, 42). Previous studies have provided conflicting results on whether tumor hormone production affects the effectiveness of adjuvant mitotane (43-45). Our results indicate that adjuvant mitotane may contribute to prolonged RFS regardless of the hormonal activity of the tumor, with no significant difference observed in OS between hormone-producing and non-hormone-producing subgroups. Therefore, this treatment can be considered irrespective of ACC hormone status.

Our findings suggest that adjuvant mitotane is associated with improved OS and RFS in patients with negative surgical margins, supporting its role in the management of this patient subgroup.

Our meta-analysis found side effects similar to previous reports, including gastrointestinal, neurological, hematological, and endocrine symptoms in mitotane-treated patients, sometimes leading to treatment discontinuation (7, 40).

The findings of studies on adjuvant radiotherapy are inconsistent (46). ACC was traditionally considered radio-resistant based on mixed results from case reports and series published until 2006 (47, 48). However, modern radiation techniques and high-quality retrospective studies have changed the perception of radiotherapy in the treatment of ACC (49).

Of the 4 previous meta-analyses that explored the impact of adjuvant radiotherapy in ACC, 3 concluded that it significantly prolongs LRFS (49-51). Of the 3 reviews that also evaluated its effectiveness in enhancing OS and RFS, 1 reported improvements in both (49), whereas the 2 others found no significant difference (39, 50).

Our results suggest that adjuvant radiotherapy has a potential clinical benefit in reducing the risk of local and distant recurrence and mortality. However, our findings were not statistically significant due to the high heterogeneity and small sample size of some studies. As indicated in previous reports, large patient cohorts are required to demonstrate a significant survival benefit from adjuvant radiation (46, 52), as individual patient outcomes can heavily influence small trials.

Current guidelines do not establish a definitive consensus on using adjuvant radiotherapy. Its use in patients with R1/Rx resection or stage III disease has been recommended only on an individual basis (36, 39). We found that adjuvant radiotherapy significantly prolonged OS and RFS in stages I to III and showed a clinical benefit in reducing the risk of mortality in stage IV disease. However, the evidence supporting its efficacy in stage IV is limited due to the low number of included studies. Several large retrospective cohorts using data from the NCDB have demonstrated its positive impact on survival in patients with R1 resection (8, 53).

According to the GRADE assessment (29), the certainty of evidence for the effect of radiotherapy on OS was rated as very low across all stages, including stage I to III and stage IV. For RFS, the certainty was moderate overall but very low within the stage I to stage III subgroup. Regarding LRFS, the certainty of evidence was rated as low. These ratings reflect the retrospective observational nature of the included studies and the lack of reported event numbers in several analyses, which limits the robustness and interpretability of the findings.

The studies included in our meta-analysis described mild, tolerable adverse effects, such as nausea, fatigue, and skin toxicities (52, 54).

Our findings suggest that radiotherapy should be considered in both stage I to III and stage IV ACC, such as those with R1 resection. Nevertheless, RCTs are necessary to confirm its efficacy.

The use of cytotoxic chemotherapy alone or with mitotane in an adjuvant setting remains a matter of debate (37). Platinum-based chemotherapy, such as EDP-M, is the standard regimen (55), and is supported by the FIRM-ACT trial (56). Current guidelines do not agree on the routine use of adjuvant cytotoxic chemotherapy; it should be considered on an individual basis in selected patients at a very high risk of recurrence (36, 39). Our analysis did not confirm the efficacy of adjuvant chemotherapy in reducing the risk of death, limited by a small patient sample and wide CIs. These findings highlight the need for robust, large-scale studies to clarify its role in the treatment of ACC. The ongoing randomized ADIUVO 2 trial, evaluating adjuvant mitotane alone vs its combination with cisplatin/etoposide after surgical resection, may provide future guidance for treating high-risk ACC patients (57). Cytotoxic chemotherapy can cause mild to severe side effects, including hematological, gastroenterological toxicity, alopecia, and fatigue, as confirmed by our reviewed studies (20, 56).

Experimental studies have shown that mitotane and radiotherapy have synergetic effects (58), confirmed by several retrospective cohorts (8, 59). Our findings indicate that adjuvant chemoradiotherapy tends to prolong OS without increasing the risk of side effects compared to radiotherapy alone (60).

In our study, we could not assess treatment strategies for pediatric ACC separately from adult cases due to the lack of data and comparative trials evaluating therapeutic efficacy in children. Current treatment protocols for pediatric ACC are primarily derived from adult management approaches (61). Surgery remains the cornerstone of therapy (62); however, in advanced stages, no standardized treatment guidelines are currently available (63). Mitotane has demonstrated benefit particularly in advanced stages, although it is poorly tolerated and requires close monitoring due to potential neurotoxicity and challenges in maintaining therapeutic levels (64). For high-risk or metastatic cases, mitotane is typically combined with chemotherapy, such as CED (cisplatin, etoposide, doxorubicin) (64), or regimens based on the German Society for Pediatric Oncology and Hematology for Malignant Endocrine Tumors (GPOH-MET) protocol (65). The use of radiotherapy in pediatric ACC remains controversial; although it may be beneficial in certain cases, its use should be carefully considered due to the high prevalence of germline TP53 mutations in this population, which substantially increases the risk of secondary malignancies (65). Targeted therapies have shown limited efficacy to date, underlining the need for improved molecular characterization and the identification of novel therapeutic targets (63). Our findings underscore the urgent need for larger, more comprehensive studies specifically focused on the pediatric population to improve evidence-based management strategies in this vulnerable group.

Our study has several strengths, including a rigorous methodology and a comprehensive analysis of key ACC treatments. Broad inclusion criteria were utilized, making the study population representative of the overall patient population and allowing for a large sample size despite the rarity of the disease. Our meta-analysis is the first to include subgroup analyses based on prognostic factors such as stage, surgical margin, and hormone secretion, and assess their impact on treatment efficacy.

To highlight the limitations, most of our findings were derived from retrospective cohorts, some of which, with small patient numbers, have the potential to introduce bias. Another limitation is the high heterogeneity among studies, which mainly results from differences in patient selection, baseline characteristics, study design, and methodological approaches. Treatments with potentially severe side effects are typically offered to patients with more serious diseases, which may cause selection bias within the studies. The lack of consistent adjustment for relevant prognostic factors led to imbalances between intervention and control groups and further contributed to the variability in treatment outcomes. These findings highlight the need for future prospective studies investigating the impact of different prognostic factors on the efficacy of various therapeutic modalities. Additionally, the analysis of the effect of mitotane on OS may be influenced by publication bias. Most of our findings were based on evidence of low or very low certainty, which further limits the strength and generalizability of our conclusions. It is important to highlight that developing appropriate treatment protocols would require evaluating the efficacy of each therapy at all stages; however, the current literature lacks adequate data to perform such an analysis.

Our findings indicate that mitotane significantly improves oncological outcomes in patients with ACC, while radiotherapy demonstrates potential advantages, especially in localized disease. Monitoring serum mitotane levels is essential for maintaining mitotane concentrations above 14 mg/L and preventing toxicity. This drug may be effective regardless of hormonal activity; however, this remains uncertain due to limited data, underscoring the need for further research. It also improves oncological outcomes in patients with negative surgical margins.

Further research is essential to evaluate the efficacy and safety of adjuvant cytotoxic chemotherapy, as well as the combined use of adjuvant mitotane and radiotherapy. Additionally, more studies are needed to analyze the effectiveness of adjuvant treatments at different stages and in pediatric patients, and to identify novel therapeutic targets to improve patient outcomes. In addition, larger RCTs are required to minimize selection bias and balance confounding factors.

In conclusion, our results highlight the positive impact of mitotane in improving recurrence-free and overall survival in the entire patient population, including those with localized tumors. Radiotherapy demonstrates potential advantages in enhancing oncological outcomes, especially for stage I to III disease. These findings suggest broader consideration of each therapy in clinical practice. However, further research is needed to assess the efficacy and safety of the combined use of these therapies. Additional research is also required to confirm the effectiveness of adjuvant cytotoxic chemotherapy, evaluate the stage-specific efficacy of radiotherapy and systemic treatments, and establish treatment protocols for childhood ACC.

The main conclusion of our study is that there is currently a lack of comprehensive and high-quality studies in the literature, which is essential to improve evidence-based treatment protocols for this aggressive malignancy. Our findings underscore the urgent need for more randomized controlled trials and well-designed, rigorous studies to support clinical decision-making and improve patient outcomes.

## Data Availability

Original data generated and analyzed during this study are included in this published article or in the data repositories listed in References.

## Abbreviations

ACC: adrenal cortical carcinoma
aHR: adjusted hazard ratio
CENTRAL: Cochrane Central Register of Controlled Trials
GRADE: Grading of Recommendations Assessment, Development, and Evaluation
HR: hazard ratio
LRFS: local recurrence-free survival
NCDB: National Cancer Database
OR: odds ratio
OS: overall survival
PRISMA: Preferred Reporting Items for Systematic Reviews and Meta-Analyses
RCT: randomized controlled trial
RFS: recurrence-free survival
SEER: Surveillance, Epidemiology, and End Results

## References

[1] Kerkhofs TM, Verhoeven RH, Van der Zwan JM, et al Adrenocortical carcinoma: a population-based study on incidence and survival in The Netherlands since 1993. Eur J Cancer. 2013;49(11):2579‐2586. 23561851 10.1016/j.ejca.2013.02.034

[2] Kebebew E, Reiff E, Duh QY, Clark OH, McMillan A. Extent of disease at presentation and outcome for adrenocortical carcinoma: have we made progress? World J Surg. 2006;30(5):872‐878. 16680602 10.1007/s00268-005-0329-x

[3] Ayala-Ramirez M, Jasim S, Feng L, et al Adrenocortical carcinoma: clinical outcomes and prognosis of 330 patients at a tertiary care center. Eur J Endocrinol. 2013;169(6):891‐899. 24086089 10.1530/EJE-13-0519 PMC4441210

[4] Tőke J, Uhlyarik A, Lohinszky J, et al Prognostic factors and mitotane treatment of adrenocortical cancer. Two decades of experience from an institutional case series. Front Endocrinol (Lausanne). 2022;13:952418. 36246926 10.3389/fendo.2022.952418 PMC9560769

[5] Chukkalore D, MacDougall K, Master V, Bilen MA, Nazha B. Adrenocortical carcinomas: molecular pathogenesis, treatment options, and emerging immunotherapy and targeted therapy approaches. Oncologist. 2024;29(9):738‐746. 38381694 10.1093/oncolo/oyae029 PMC11379653

[6] Tierney JF, Chivukula SV, Poirier J, et al National treatment practice for adrenocortical carcinoma: have they changed and have we made any progress? J Clin Endocrinol Metab. 2019;104(12):5948‐5956. 31361313 10.1210/jc.2019-00915

[7] Pan LH, Yen CC, Huang CJ, Ng XN, Lin LY. Prognostic predictors of adrenocortical carcinoma: a single-center thirty-year experience. Front Endocrinol (Lausanne). 2023;14:1134643. 36967802 10.3389/fendo.2023.1134643 PMC10036850

[8] Hickey K, Shakir A, Shepherd C, Djang R, Patel S. Impact of multimodal therapy on margin status on overall survival for patients undergoing adrenalectomy for localized adrenocortical carcinoma. Indian J Urol. 2022;38(4):276‐281. 36568465 10.4103/iju.iju_77_22 PMC9787443

[9] Amini N, Margonis GA, Kim Y, et al Curative resection of adrenocortical carcinoma: rates and patterns of postoperative recurrence. Ann Surg Oncol. 2016;23(1):126‐133. 26282907 10.1245/s10434-015-4810-y PMC4962540

[10] Glenn JA, Else T, Hughes DT, et al Longitudinal patterns of recurrence in patients with adrenocortical carcinoma. Surgery. 2019;165(1):186‐195. 30343951 10.1016/j.surg.2018.04.068

[11] Fay AP, Elfiky A, Teló GH, et al Adrenocortical carcinoma: the management of metastatic disease. Crit Rev Oncol Hematol. 2014;92(2):123‐132. 24958272 10.1016/j.critrevonc.2014.05.009 PMC4578298

[12] Wu K, Liu X, Liu Z, Lu Y, Wang X, Li X. Benefit of postoperative radiotherapy for patients with nonmetastatic adrenocortical carcinoma: a population-based analysis. J Natl Compr Canc Netw. 2021;19(12):1425‐1432. 34902831 10.6004/jnccn.2021.7035

[13] Shariq OA, McKenzie TJ. Adrenocortical carcinoma: current state of the art, ongoing controversies, and future directions in diagnosis and treatment. Ther Adv Chronic Dis. 2021;12:20406223211033103. 34349894 10.1177/20406223211033103 PMC8295938

[14] Netto AD, Wajchenberg BL, Ravaglia C, et al Treatment of adrenocortical cancer with O,P'-DDD. Ann Intern Med. 1963;59(1_Part_1):74‐78. 14042612 10.7326/0003-4819-59-1-74

[15] Bergenstal DM, Hertz R, Lipsett MB, Moy RH. Chemotherapy of adrenocortical cancer with O,P’DDD. Ann Intern Med. 1960;53(4):672‐682.

[16] Puglisi S, Calabrese A, Basile V, et al New perspectives for mitotane treatment of adrenocortical carcinoma. Best Pract Res Clin Endocrinol Metab. 2020;34(3):101415. 32179008 10.1016/j.beem.2020.101415

[17] Paragliola RM, Torino F, Papi G, Locantore P, Pontecorvi A, Corsello SM. Role of mitotane in adrenocortical carcinoma—review and state of the art. Eur Endocrinol. 2018;14(2):62‐66. 30349596 10.17925/EE.2018.14.2.62 PMC6182924

[18] Basile V, Puglisi S, Calabrese A, et al Unwanted hormonal and metabolic effects of postoperative adjuvant mitotane treatment for adrenocortical cancer. Cancers (Basel). 2020;12(9):2615. 32937772 10.3390/cancers12092615 PMC7565701

[19] Bianchini M, Puliani G, Chiefari A, Mormando M, Lauretta R, Appetecchia M. Metabolic and endocrine toxicities of mitotane: a systematic review. Cancers (Basel). 2021;13(19):5001. 34638485 10.3390/cancers13195001 PMC8508479

[20] Kimpel O, Bedrose S, Megerle F, et al Adjuvant platinum-based chemotherapy in radically resected adrenocortical carcinoma: a cohort study. Br J Cancer. 2021;125(9):1233‐1238. 34400803 10.1038/s41416-021-01513-8 PMC8548516

[21] de Padua TC, Marandino L, Raggi D, et al A systematic review of published clinical trials in the systemic treatment of adrenocortical carcinoma: an Initiative Led on Behalf of the Global Society of Rare Genitourinary Tumors. Clin Genitourin Cancer. 2023;21(1):1‐7. 36376169 10.1016/j.clgc.2022.10.011

[22] Pfeffer A, Beke N, Bakó D, et al Data from: Supplementary Material for “Efficacy and Safety of Radiotherapy and Systemic Treatments in Adrenocortical Carcinoma: Systematic Review and Meta-Analysis”. Zenodo Repository. 2025. Deposited 26 August 2025. 10.5281/zenodo.15882187 PMC12623036 40796146

[23] Page MJ, McKenzie JE, Bossuyt PM, et al The PRISMA 2020 statement: an updated guideline for reporting systematic reviews. BMJ. 2021;372:n71. 33782057 10.1136/bmj.n71 PMC8005924

[24] Higgins J, Thomas J, Chandler J, et al, editors. Cochrane handbook for systematic reviews of interventions version 6.3 (updated February 2022). www.training.cochrane.org/handbook

[25] Haddaway NR, Grainger MJ, Gray CT. Citationchaser: a tool for transparent and efficient forward and backward citation chasing in systematic searching. Res Synth Methods. 2022;13(4):533‐545. 35472127 10.1002/jrsm.1563

[26] Sterne JAC, Savović J, Page MJ, et al Rob 2: a revised tool for assessing risk of bias in randomised trials. BMJ. 2019;366:l4898. 31462531 10.1136/bmj.l4898

[27] Sterne JA, Hernán MA, Reeves BC, et al ROBINS-I: a tool for assessing risk of bias in non-randomised studies of interventions. BMJ. 2016;355:i4919. 27733354 10.1136/bmj.i4919 PMC5062054

[28] Hayden JA, van der Windt DA, Cartwright JL, Côté P, Bombardier C. Assessing bias in studies of prognostic factors. Ann Intern Med. 2013;158(4):280‐286. 23420236 10.7326/0003-4819-158-4-201302190-00009

[29] Guyatt G, Oxman AD, Akl EA, et al GRADE guidelines: 1. Introduction-GRADE evidence profiles and summary of findings tables. J Clin Epidemiol. 2011;64(4):383‐394. 21195583 10.1016/j.jclinepi.2010.04.026

[30] Renehan AG, Zwahlen M, Minder C, O'Dwyer ST, Shalet SM, Egger M. Insulin-like growth factor (IGF)-I, IGF binding protein-3, and cancer risk: systematic review and meta-regression analysis. Lancet. 2004;363(9418):1346‐1353. 15110491 10.1016/S0140-6736(04)16044-3

[31] *WebPlotDigitizer*. Version 5.1. 2011. Accessed July 29, 2024. https://automeris.io

[32] Higgins J, Thomas J, Chandler J, et al, editors. Cochrane handbook for systematic reviews of interventions version 6.5. Updated (August 2024). www.training.cochrane.org/handbook

[33] Haddaway NR, Page MJ, Pritchard CC, McGuinness LA. PRISMA2020: an R package and Shiny app for producing PRISMA 2020-compliant flow diagrams, with interactivity for optimised digital transparency and Open Synthesis. Campbell Syst Rev. 2022;18(2):e1230. 36911350 10.1002/cl2.1230 PMC8958186

[34] Hegyi P, Petersen OH, Holgate S, et al Academia Europaea Position Paper on Translational Medicine: the cycle model for translating scientific results into community benefits. J Clin Med. 2020;9(5):1532. 32438747 10.3390/jcm9051532 PMC7290380

[35] Hegyi P, Erőss B, Izbéki F, Párniczky A, Szentesi A. Accelerating the translational medicine cycle: the Academia Europaea pilot. Nat Med. 2021;27(8):1317‐1319. 34312557 10.1038/s41591-021-01458-8

[36] Fassnacht M, Assie G, Baudin E, et al Adrenocortical carcinomas and malignant phaeochromocytomas: ESMO-EURACAN Clinical Practice Guidelines for diagnosis, treatment and follow-up. Ann Oncol. 2020;31(11):1476‐1490. 32861807 10.1016/j.annonc.2020.08.2099

[37] Bedrose S, Daher M, Altameemi L, Habra MA. Adjuvant therapy in adrenocortical carcinoma: reflections and future directions. Cancers (Basel). 2020;12(2):508. 32098326 10.3390/cancers12020508 PMC7072549

[38] Tang Y, Liu Z, Zou Z, Liang J, Lu Y, Zhu Y. Benefits of adjuvant mitotane after resection of adrenocortical carcinoma: a systematic review and meta-analysis. Biomed Res Int. 2018;2018:9362108. 29967789 10.1155/2018/9362108 PMC6008618

[39] Fassnacht M, Dekkers OM, Else T, et al European Society of Endocrinology Clinical Practice Guidelines on the management of adrenocortical carcinoma in adults, in collaboration with the European Network for the Study of Adrenal Tumors. Eur J Endocrinol. 2018;179(4):G1‐G46. 30299884 10.1530/EJE-18-0608

[40] Terzolo M, Fassnacht M, Perotti P, et al Adjuvant mitotane versus surveillance in low-grade, localised adrenocortical carcinoma (ADIUVO): an international, multicentre, open-label, randomised, phase 3 trial and observational study. Lancet Diabetes Endocrinol. 2023;11(10):720‐730. 37619579 10.1016/S2213-8587(23)00193-6 PMC10522778

[41] Sada A, Foster TR, Al-Ward R, et al The effect of hormonal secretion on survival in adrenocortical carcinoma: a multi-center study. Surgery. 2024;175(1):80‐89. 37945477 10.1016/j.surg.2023.04.070

[42] Vanbrabant T, Fassnacht M, Assie G, Dekkers OM. Influence of hormonal functional status on survival in adrenocortical carcinoma: systematic review and meta-analysis. Eur J Endocrinol. 2018;179(6):429‐436. 30325179 10.1530/EJE-18-0450

[43] Abiven G, Coste J, Groussin L, et al Clinical and biological features in the prognosis of adrenocortical cancer: poor outcome of cortisol-secreting tumors in a series of 202 consecutive patients. J Clin Endocrinol Metab. 2006;91(7):2650‐2655. 16670169 10.1210/jc.2005-2730

[44] Bertherat J, Coste J, Bertagna X. Adjuvant mitotane in adrenocortical carcinoma. N Engl J Med. 2007;357(12):1256‐1257; author reply 1259. 17881760 10.1056/NEJMc076267

[45] Margonis GA, Kim Y, Tran TB, et al Outcomes after resection of cortisol-secreting adrenocortical carcinoma. Am J Surg. 2016;211(6):1106‐1113. 26810939 10.1016/j.amjsurg.2015.09.020 PMC4957943

[46] Scollo C, Russo M, Trovato MA, et al Prognostic factors for adrenocortical carcinoma outcomes. Front Endocrinol (Lausanne). 2016;7:99. 27504106 10.3389/fendo.2016.00099 PMC4958635

[47] Luton JP, Cerdas S, Billaud L, et al Clinical features of adrenocortical carcinoma, prognostic factors, and the effect of mitotane therapy. N Engl J Med. 1990;322(17):1195‐1201. 2325710 10.1056/NEJM199004263221705

[48] Bodie B, Novick AC, Pontes JE, et al The Cleveland Clinic experience with adrenal cortical carcinoma. J Urol. 1989;141(2):257‐260. 2913342 10.1016/s0022-5347(17)40734-8

[49] Zhu J, Zheng Z, Shen J, et al Efficacy of adjuvant radiotherapy for treatment of adrenocortical carcinoma: a retrospective study and an updated meta-analysis. Radiat Oncol. 2020;15(1):118. 32448148 10.1186/s13014-020-01533-3 PMC7245885

[50] Viani GA, Viana BS. Adjuvant radiotherapy after surgical resection for adrenocortical carcinoma: a systematic review of observational studies and meta-analysis. J Cancer Res Ther. 2019;15(Suppl 1):S20‐S26. 30900615 10.4103/jcrt.JCRT_996_15

[51] Srougi V, de Bessa J, Tanno J, et al Adjuvant radiotherapy for the primary treatment of adrenocortical carcinoma: are we offering the best? Int Braz J Urol. 2017;43(5):841‐848. 28727379 10.1590/S1677-5538.IBJU.2017.0095 PMC5678514

[52] Sabolch A, Else T, Griffith KA, et al Adjuvant radiation therapy improves local control after surgical resection in patients with localized adrenocortical carcinoma. Int J Radiat Oncol Biol Phys. 2015;92(2):252‐259. 25754631 10.1016/j.ijrobp.2015.01.007

[53] Nelson DW, Chang SC, Bandera BC, Fischer TD, Wollman R, Goldfarb M. Adjuvant radiation is associated with improved survival for select patients with non-metastatic adrenocortical carcinoma. Ann Surg Oncol. 2018;25(7):2060‐2066. 29748889 10.1245/s10434-018-6510-x

[54] Fassnacht M, Hahner S, Polat B, et al Efficacy of adjuvant radiotherapy of the tumor bed on local recurrence of adrenocortical carcinoma. J Clin Endocrinol Metab. 2006;91(11):4501‐4504. 16895957 10.1210/jc.2006-1007

[55] Laganà M, Grisanti S, Cosentini D, et al Efficacy of the EDP-M scheme plus adjunctive surgery in the management of patients with advanced adrenocortical carcinoma: the Brescia experience. Cancers (Basel). 2020;12(4):941. 32290298 10.3390/cancers12040941 PMC7226395

[56] Fassnacht M, Terzolo M, Allolio B, et al Combination chemotherapy in advanced adrenocortical carcinoma. N Engl J Med. 2012;366(23):2189‐2197. 22551107 10.1056/NEJMoa1200966

[57] Sarvestani AL, Gregory SN, Teke ME, et al Mitotane with or without cisplatin and etoposide for patients with a high risk of recurrence in stages 1-3 adrenocortical cancer after surgery. Ann Surg Oncol. 2023;30(2):680‐682. 36305989 10.1245/s10434-022-12725-4

[58] Cerquetti L, Sampaoli C, Amendola D, et al Mitotane sensitizes adrenocortical cancer cells to ionizing radiations by involvement of the cyclin B1/CDK complex in G2 arrest and mismatch repair enzymes modulation. Int J Oncol. 2010;37(2):493‐501. 20596677 10.3892/ijo_00000698

[59] Skertich NJ, Tierney JF, Chivukula SV, et al Risk factors associated with positive resection margins in patients with adrenocortical carcinoma. Am J Surg. 2020;220(4):932‐937. 32111342 10.1016/j.amjsurg.2020.02.043

[60] Sabolch A, Else T, Williams A, et al Toxicity of concurrent radiation therapy with mitotane compared to radiation therapy alone in the adjuvant treatment of adrenocortical carcinoma. Int J Radiat Oncol Biol Phys. 2014;90(1):S466‐S467.

[61] Ilanchezhian M, Varghese DG, Glod JW, et al Pediatric adrenocortical carcinoma. Front Endocrinol (Lausanne). 2022;13:961650. 36387865 10.3389/fendo.2022.961650 PMC9659577

[62] Hubertus J, Boxberger N, Redlich A, von Schweinitz D, Vorwerk P. Surgical aspects in the treatment of adrenocortical carcinomas in children: data of the GPOH-MET 97 trial. Klin Padiatr. 2012;224(3):143‐147. 22504769 10.1055/s-0032-1304627

[63] Virgone C, Roganovic J, Vorwerk P, et al Adrenocortical tumours in children and adolescents: the EXPeRT/PARTNER diagnostic and therapeutic recommendations. Pediatr Blood Cancer. 2021;68(S4):e29025. 34174161 10.1002/pbc.29025

[64] Rodriguez-Galindo C, Krailo MD, Pinto EM, et al Treatment of pediatric adrenocortical carcinoma with surgery, retroperitoneal lymph node dissection, and chemotherapy: the Children's Oncology Group ARAR0332 Protocol. J Clin Oncol. 2021;39(22):2463‐2473. 33822640 10.1200/JCO.20.02871 PMC8462560

[65] Redlich A, Boxberger N, Strugala D, et al Systemic treatment of adrenocortical carcinoma in children: data from the German GPOH-MET 97 trial. Klin Padiatr. 2012;224(6):366‐371. 23143764 10.1055/s-0032-1327579

